# Le nerf laryngé inférieur: considérations anatomiques et chirurgicales à propos de 60 thyroïdectomies

**DOI:** 10.11604/pamj.2019.33.58.14271

**Published:** 2019-05-27

**Authors:** Bouchaib Hemmaoui, El Arbi Bouaiti, Mohamed Sahli, Noureddine Errami, Mohamed Moumni, Ilias Benchafai, Ali Jahidi, Mohamed Zalagh, Fouad Benariba

**Affiliations:** 1 Service ORL et CCF, Hôpital Militaire Mohamed V Rabat, Faculté de Médecine et de Pharmacie, Université Mohamed V, Rabat, Maroc; 2 Laboratoire de Biostatistique, de Recherche Clinique et d’Epidémiologie, Faculté de Médecine et de Pharmacie, Université Mohamed V Rabat, Maroc

**Keywords:** Nerf laryngé inferieur, thyroïde, chirurgie, Inferior laryngeal nerve, thyroïd, surgery

## Abstract

La chirurgie thyroïdienne nécessite une parfaite connaissance de l'anatomie cervicale et les variations anatomiques, notamment celui du nerf laryngé inferieur afin d'éviter des lésions iatrogènes. L'objectif de notre étude était d'analyser les rapports du nerf laryngé inférieur, l'existence de branches de division nerveuses et une appréciation subjective du calibre du nerf. Il s'agit d'une étude prospective sur 1 an incluant 60 patients ayant bénéficié d'une thyroïdectomie. Soixante patients ont bénéficié d'une chirurgie sur la glande thyroïde entre février 2014 et janvier 2015 par le même opérateur principal (10 hommes et 50 femmes). L'âge moyen de nos patients était de 51 ans. Pour les hommes ont été réalisées 6 thyroïdectomies totales, 2 lobo-isthmectomies gauches et 2 lobo-isthmectomies droites. Pour les femmes ont été réalisées 33 thyroïdectomies totales, 8 lobo-isthmectomies gauches et 9 lobo-isthmectomies droites. À droite, le nerf était superficiel par rapport à l'artère dans 71,6% des cas, il était divisé dans 33,3% des cas et il était anormalement fin dans 16,6% des cas. À gauche, le nerf était profond par rapport à l'artère dans 83,3% des cas, il était divisé dans 15% des cas et il était anormalement fin dans 11,6% des cas. La connaissance des variations anatomiques du nerf laryngé inferieur est indispensable dans la chirurgie thyroïdienne, le risque est particulièrement important du côté droit vu le calibre parfois très petit et l'existence de branches division plus fréquente que du côté gauche.

## Abstract (en)

Thyroid surgery requires a thorough knowledge of cervical anatomy and anatomical variations, in particular of the lower laryngeal nerve, in order to avoid iatrogenic lesions. The objective of our study was to analyze the relationships of the lower laryngeal nerve, the existence of branches of nerve division and a subjective appreciation of the size of the nerve. This is a prospective study of 1 year including 60 patients who underwent thyroidectomy. Sixty patients underwent surgery on the thyroid gland between February 2014 and January 2015 by the same principal operator (10 men and 50 women). The average age of our patients was 51 years. For men were performed 6 total thyroidectomies, 2 left lobo-isthmectomies and 2 right lobo-isthmectomies. For women were performed 33 total thyroidectomies, 8 left lobo-isthmectomies and 9 right lobo-isthmectomies. On the right, the nerve was superficial relative to the artery in 71.6% of cases; it was divided in 33.3% of cases and was abnormally thin in 16.6% of cases. On the left, the nerve was deep in relation to the artery in 83.3% of cases; it was divided in 15% of cases and was abnormally thin in 11.6% of cases. Knowledge of the anatomical variations of the lower laryngeal nerve is essential in thyroid surgery, the risk is particularly important on the right side given the sometimes very small caliber and the existence of branches of division more frequent than on the left side.

## Introduction

La chirurgie thyroïdienne est l'une des plus pratiquées en chirurgie cervico-faciale, de ce fait la connaissance de l'anatomie cervicale est particulièrement importante pour éviter les complications, notamment les lésions du nerf laryngé inférieur (NLI). La dissection peropératoire du NLI est actuellement admise par un grand nombre d'auteurs et permet également de réduire le pourcentage de lésions iatrogènes [1]. La lésion du NLI est une complication iatrogène responsable de troubles importants de la respiration et de la déglutition d'où l'importance de bien connaître son anatomie de son origine à sa terminaison et de faire un repérage et une dissection prudente à son contact. L'objectif de notre travail est d'étudier les rapports du NLI et ses variations anatomiques.

## Méthodes

Il s'agit d'une étude prospective sur 18 mois (mai 2014 / décembre 2015). 60 patients ont bénéficié d'une intervention chirurgicale sur la glande thyroïde par la même équipe chirurgicale. Au cours de l'acte chirurgical, le NLI était exposé sur toute sa longueur dans la loge thyroïdienne, on a étudié les rapports du nerf avec les branches de l'artère thyroïdienne inferieure (ATI), l'existence de branches de division nerveuses extra-laryngées et on a fait une appréciation subjective de son calibre. Tous les patients avaient eu un examen ORL (oto-rhino-laryngologie) préopératoire systématique à la nasofibroscopie permettant d'éliminer une paralysie récurrentielle préopératoire secondaire à la pathologie thyroïdienne ou en rapport avec une autre cause.

## Résultats

L'âge moyen de nos patients était de 51 ans et le sex ratio était de 0,2 (10 hommes et 50 femmes). Nous avons repéré un total de 99 NLI dont 50 à droite et 49 à gauche. Aucun nerf laryngé inférieur non récurrent n'a été mis en évidence. Leurs rapports s'effectuaient surtout avec les branches de l'artère thyroïdienne inférieure: à droite, le nerf était superficiel par rapport à l'artère dans 72% des cas (36 cas), il était divisé dans 32% des cas (16 cas) et il était anormalement fin dans 16% des cas (8 cas); à gauche, le nerf était profond par rapport à l'artère dans 83,3% des cas (41 cas), il était divisé dans 14,28% des cas (7 cas) et il était anormalement fin dans 10,2% des cas (5 cas).Le trajet de 93% des nerfs disséqués était postérolatéral au ligament de Grüber. Ces données sont résumées dans le Tableau 1.

**Tableau 1:** Rapports et variations anatomiques du nerf laryngé inférieur (NLI) dans notre série

	Localisation / ATI	Bifurcation	Calibre: Anormalement fin
*A droite*	Superficiel **72%** des cas	**32%**	**16%**
*A gauche*	Profond **83,67%** des cas	**14,28%**	**10,20%**

## Discussion

La chirurgie thyroïdienne est considérée actuellement comme un pilier important dans le traitement de multiples pathologies thyroïdiennes. Cette chirurgie nécessite toutefois l'association de plusieurs compétences multidisciplinaires; endocrinologue, chirurgien, radiologue, oncologue pour une meilleure prise en charge du patient. Ses indications s'élargissent de plus en plus et intéressent aussi la pathologie maligne que bénigne. Cependant la région thyroïdienne est riche en organes nobles ce qui confère à l'acte chirurgical des risques de complications vitales et fonctionnelles non négligeables et impose une connaissance parfaite et une bonne maîtrise de la pathologie et de l'anatomie cervicale notamment celle du NLI [2]. Auparavant, cette chirurgie thyroïdienne ne se basait pas sur une dissection systématique du NLI, pour éviter un traumatisme de ce dernier les chirurgiens préféraient soit laisser un mur postérieur de sécurité soit de rester au contact avec la capsule thyroïdienne. Actuellement, la tendance semble être une dissection systématique [1].

Croisement du NLI et l'ATI: de nombreuses variations de situation du NLI par rapport à l'ATI ont été décrites, selon une étude multicentrique réalisée par Nyeki sur 62 dissections [3], les trajets du NLI étaient souvent rétrovasculaires, dans 53,1% à droite et 76,6% à gauche. Dans 15,6% et 13,4%, respectivement à droite et à gauche, le trajet nerveux était transvasculaire. Echeverria Monares [4], a réalisé une étude anatomique très précise sur les rapports entre le nerf laryngé inférieur et l'artère thyroïdienne inférieure, schématiquement la position pré-artérielle domine à droite, la position rétro-artérielle, à gauche. Néanmoins toutes les positions sont possibles à droite comme à gauche. II est rétro-artériel dans 47% des cas, pré-artériel dans 28% des cas, inter-artériel dans 25% des cas. Nos résultats sur ce sujet sont concordants avec les données de la littérature: à droite le nerf était superficiel par rapport à l'artère dans environ deux tiers des ca alors qu'à gauche il était plus constamment profond (90% des cas). Ces résultats sont résumés dans le Tableau 2 [3, 5].

**Tableau 2:** Situation du NLI par rapport à l’artère thyroïdienne inferieure (ATI) et les principales données concernant la division du nerf laryngé inférieur (NLI)

	AN Neyki et al [3] (62 dissections)	C Page et al [5] (205 dissections)	Notre étude
A DROITE	Superficiel 53,1%	Superficiel 66,83%	Superficiel 76%
A GAUCHE	Profond 76,6%	Profond 88,89%	Profond 83,67%
	**AN Neyki et al [****3****] (62 dissections)**	**Page et al [****5****] (251 dissections)**	**Notre étude**
Bifurcation à droite	9,7%	23,41%	32%
Bifurcation à gauche		15,15%	14,28%

Rapport avec le ligament de Gruber: c'est à la partie terminale de son trajet extralaryngé que le nerf récurrent contracte des rapports intimes avec la glande. Il est souvent situé juste au-dessous de la zone d'adhérence glandulaire à la trachée: c'est le classique ligament de Gruber et Sappey ou lame thyrotrachéale transverse, condensation de tissu conjonctif vasculaire amarrant solidement la partie postéro-interne des lobes thyroïdiens aux deux premiers anneaux trachéaux [2]. La description classique veut que le NLI traverse latéralement le ligament dans environ 88%, mais dans 12% des cas, le nerf peut cheminer à travers le ligament lui-même [6]. Dans notre étude le NLI était latéral par rapport au ligament dans 93%. Ces rapports intimes expliquent le fait que cette zone est la plus difficile région pour la dissection du NLI [7, 8].

Division terminale du NLI

La pénétration du nerf récurrent dans le larynx constitue un repère fiable. La pénétration laryngée du nerf se fait sous l'arcade inférieure du constricteur inférieur et en arrière de la corne inférieure du cartilage thyroïde. Le dédoublement du nerf est un cas de figure anatomique rare, constituant alors un piège anatomique pouvant faire prendre l'une des branches pour le tronc lui-même et négliger l'autre. La division précoce du nerf avant sa pénétration laryngée est donc une éventualité qu'on doit prendre en considération. D'après Echeverria Monares, le récurrent peut se présenter, par ordre de fréquence décroissant: comme un tronc unique; dédoublé en forme de V; trifurqué; ou enfin plexiforme. Les données de la littérature sont variables voire divergentes, mais l'existence de deux ou trois branches extra-laryngées n'est pas rare, en particulier à droite. Le segment terminal du NLI (situé à 35mm au-dessous du bord inferieur du constricteur inferieur du pharynx) présente une division extra laryngée dans 65 à 92% [9]. Les fibres motrices sont en général localisées dans les branches antérieures de la division extra laryngée [10]. Dans une série de Page sur 251 thyroïdectomies, le NLI était divisé dans près d'un quart des cas à droite (23,41%) et environ 15% à gauche. Le Tableau 2 [3, 5] résume les principales données concernant la division du NLI.

Calibre du NLI: il faut en général toujours garder à l'esprit que le NLI possède un calibre relativement important, mais parfois le NLI peut être fin et grêle posant ainsi un problème lors de son repérage et favorisant ainsi son traumatisme per opératoire. Dans notre étude, le NLI était fin dans 16% à droite et dans 10,20% à gauche Une différence de calibre selon le sexe a été notée dans l'étude de Page *et al.*, montrant que la fréquence de ces nerfs grêles était environ 5% pour les hommes contre 14% chez les femmes à droite, et environ 2% chez les hommes contre 11% chez les femmes à gauche [5].

## Conclusion

Les rapports du NLI avec l'ATI sont très variables rendant ainsi difficile de considérer cette artère comme repère unique pour la dissection du nerf récurrent. En plus, la pathologie thyroïdienne qui modifie considérablement le trajet du nerf laryngé et la présence de branches extralaryngées représente des variations anatomiques rendant délicates la dissection récurrentielle. Le calibre du NLI joue également un rôle important dans le repérage de celui-ci, un calibre plus grêle est décrit plus chez les femmes et favorise sa lésion iatrogène. Enfin, de nombreux auteurs ont recommandé la pratique d'un «monitoring» peropératoire du nerf récurrent permettant un repérage visuel et électrique du nerf récurrent [11, 12].

### État des connaissances actuelles sur le sujet

- Les indications de la pathologie thyroïdienne sont devenues très fréquentes;
- Le risque de lésion du nerf laryngé inferieur est important;
- Beaucoup de médico-légal à la suite de toute blessure du nerf laryngé inferieur.

### Contribution de notre étude à la connaissance

- La tendance actuelle dans la chirurgie thyroïdienne est la dissection systématique du nerf laryngé inferieur pour minimiser le risque de traumatisme du nerf;
- La zone est la plus difficile dans la dissection du nerf laryngé inferieur est son rapport avec le ligament de Gruber;
- Les auteurs recommandent actuellement la pratique d'un « monitoring » peropératoire du nerf récurrent permettant un repérage visuel et électrique du nerf récurrent.

## Conflits des intérêts

Les auteurs ne déclarent aucun conflit d'intérêts.
